# A Rare Occurrence of an Extensive Sino-Nasal Solitary Fibrous Tumour

**DOI:** 10.22038/IJORL.2022.58819.3032

**Published:** 2022-07

**Authors:** Pei Fen Cheah, Azman Ali, Jothi Shanmuganathan

**Affiliations:** 1 *Department of Otorhinolaryngology, Hospital Sultanah Aminah, Johor Bahru, Johor, Malaysia.*; 2 *Department of Otorhinolaryngology, University of Malaya Medical Centre, Kuala Lumpur, Malaysia.*

**Keywords:** Solitary Fibrous Tumors, Sinonasal, Base of Skull, Orbit, Surgical Resection

## Abstract

**Introduction:**

Solitary fibrous tumours are uncommon in head and neck region, especially in the nasal cavities and paranasal sinuses, with most cases reported in the thoracic region in the pleura. It is often considered a borderline or low-grade malignant soft tissue tumour. Complete surgical resection is currently the treatment of choice, though intracranial and orbital extension of these lesions must be carefully evaluated and navigated to ensure a safe outcome.

**Case Report:**

A 36 years-old lady presented with a long one-year history of left-sided nasal obstruction with facial pain, headaches and mild visual disturbances. She had been treated for sinusitis for a prolonged period. Clinically, there was a left nasal mass obliterating the ostiomeatal complexes and the roof of the nasal cavity. MRI showed heterogeneously enhancing mass occupying the left ethmoid sinuses extending laterally eroding the left lamina papyracea to the orbit, medially towards the right nasal cavity eroding the nasal septum, and superiorly to extend intracranially. After inconclusive biopsies were performed, the mass was excised with a combined endoscopic and open lateral rhinotomy approach with left medial maxillectomy and reconstruction of the skull base defect. The tumour was eventually reported as a solitary fibrous tumour.

**Conclusions:**

Solitary fibrous tumour is a rare differential of tumours in the sino-nasal region, diagnosed via histopathology. Although generally slow-growing, these lesions may extend the adjacent structures namely the orbit and skull base. Definitive treatment via surgical resection may be performed safely after careful radiological assessment and multidisciplinary consideration.

## Introduction

Solitary fibrous tumours (SFTs) are a rare type of spindle cell neoplasm which is mesenchymal in origin. Its histogenesis is controversial, whether its origin is mesothelial or undifferentiated mesenchymal cell (1). The majority of solitary fibrous tumours occur in the thorax, predominantly at the pleura. Nevertheless, extrapleural cases had been reported, such as in the abdomen, pelvis, extremities, head and neck regions (2). 6% of these tumours are reportedly present in the head and neck region (2). However, solitary fibrous tumours of nasal cavities and paranasal sinuses are extremely rare, with less than 0.1% of all sino-nasal neoplasms. According to WHO Classification of Head and Neck Tumours 2017, solitary fibrous tumour of the nasal cavity and paranasal sinuses is classified as borderline or low-grade malignant soft tissue tumours. Most of the sino-nasal solitary fibrous tumours exhibit benign behaviour. Thus, the gold standard of treatment is complete surgical excision and it is usually curative (3). In this case, we describe a case of solitary fibrous tumour of the left ethmoid sinus which demonstrated significant local extension with skull base erosion and orbital extension.

## Case Report

**Fig 1:**
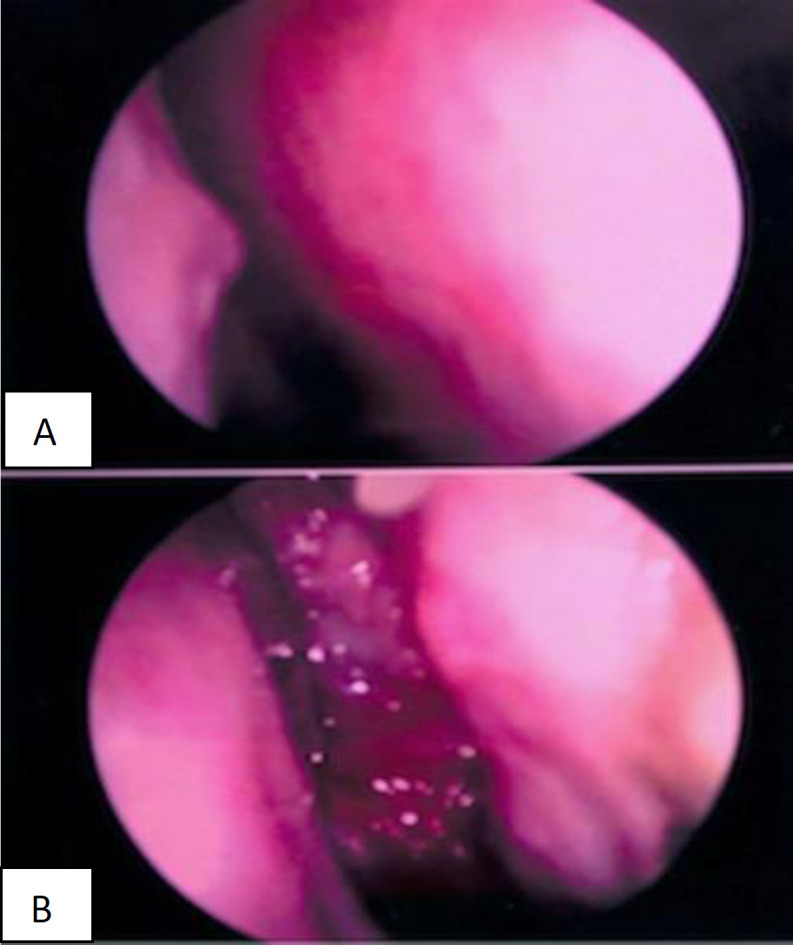
Clinical photograph of the endoscopic view of the left sino-nasal solitary fibrous tumour

A 36 years-old lady, of Indian descent, presented to our clinic in September 2019 with a complaint of chronic left-sided nasal obstruction for one year. This was associated with ipsilateral headache and intermittent facial pain, with mucoid rhinorrhea and anosmia. She also complained of epiphora of the left eye and mild visual disturbances. She had frequented her local practitioner and was regularly treated for chronic rhinosinusitis. Upon review, there was an inapparent asymmetry of the left nasal bridge with fullness medial to the left medial canthus. On nasoendoscopy, there was a large left nasal mass arising from superiorly, occupying nearly the entire left nasal cavity (Figure 1). A 4mm scope managed to bypass the mass along the floor to visualize a normal-appearing nasopharynx showing no extension of the mass inferiorly to the oropharynx. The right nasal cavity was narrowed with a significantly deviated nasal septum, likely due to mass effect. Otherwise, there were no enlarged neck nodes. The visual acuity was 6/9 bilaterally with no papilloedema. There was no ophthalmoplegia or other cranial neuropathies. 

**Fig 2:**
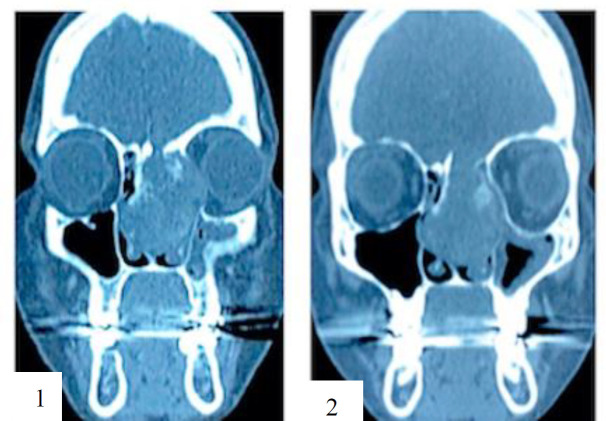
Computed Tomography of PNS (coronal) showing bony erosion through (1) lamina papyracea and (2) skull base

The patient had earlier performed computed tomography (CT) of the paranasal sinus which reported an extensive lobular mass along the left lateral nasal wall predominantly centred in the middle meatal region with breeching and destruction of the medial wall of the left maxillary sinus and possible extension into it, with soft tissues densities occupying the ethmoid and frontal sinuses, extending superiorly with breaching of the skull base and laterally eroding the lamina papyracea into the extraconal orbit. There were also soft tissue densities occupying the left sphenoid sinus (Figure 2).

**Fig 3:**
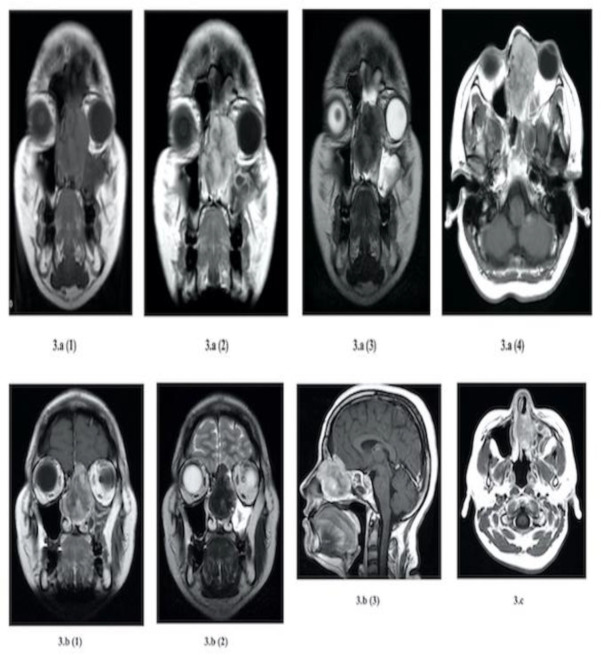
MRI Brain and PNS images of mass extensions into Orbit and Intracranially (extradural)

In view of the extension into the anterior skull base, the patient was further evaluated with magnetic resonance imaging (MRI) of the brain. MRI brain revealed the presence of heterogeneously enhancing mass measuring 4.7x4.9x3.5cm (CCxAPxW) occupying the left ethmoid sinuses extended medially to both left and right nasal cavity with obliteration of nasal septum noted. The left lamina papyracea was eroded by the mass with extension to the medial part of the extraconal space of the left orbit compressing the left medial rectus, with no abnormal enhancement. The mass also extended superiorly to the left frontal sinus and erosion of frontal bone was seen with intracranial extension to the left frontal region with mass effect into the left frontal cortex and focal thickening of intact dura in the region. There was no abnormal enhancement or focal lesion of the left frontal lobe or other intraparenchymal lesions. There was mucosal thickening in the left maxillary and sphenoid sinuses (Figure 3). The imaging performed suggested a possibility of inverted papilloma with intracranial extra-axial extension. 

- Lateral extension into orbit, extraconal, (1) isointense on T1W, (2) hypointense on T2W, (3) heterogeneous enhancement post-gadolinium contrast, (4) axial section.
- Superior extension intracranially, extra-axial. (1) T2W, (2) T1W+Gado, (3) sagittal section.
- Medial extension to contralateral nose with septal erosion.

The decision was made for a biopsy of the left nasal mass to be performed in the clinic for histopathological evaluation. This was performed twice, over a week, however, with both samples yielding only granulation tissue. No deeper biopsies were performed due to significant bleeding during the procedure which was controlled with compression.

The decision for surgical intervention endonasally, was made after further discussions and consultations with the local neurosurgical team. The patient was subjected to a left medial maxillectomy and resection of the left sino-nasal mass via a combined endoscopic and open (lateral rhinotomy) approach, with subsequent reconstruction. No peri-operative embolization was performed. Intra-operative findings showed a large firm, lobulated mass occupying the entire left nasal cavity and partially extending into the left maxillary sinus and bulging into the medial wall of the left orbit with a severely thinned out lamina papyracea. The tumour was excised en bloc with no significant bleeding encountered. A significant posterosuperior septal perforation was noted. There was a presence of skull base defect located posteromedial to the left frontal sinus opening, measuring 2cm x 1cm. After complete resection of the tumour, the dura was inspected and appeared to be intact. The skull base defect was reconstructed using a segment of fresh septal cartilage and mucosal flap from the lower left lateral wall of the nose pedicled to the posterior stump of the inferior turbinate. Polypoidal mucosae were also removed from the maxillary and sphenoid sinuses. The left lacrimal sac was marsupialized. Post-operatively the nasal cavity was packed in BIPP impregnated nasal packing which was removed after a week. The post-operative period was uneventful.

**Fig 4:**
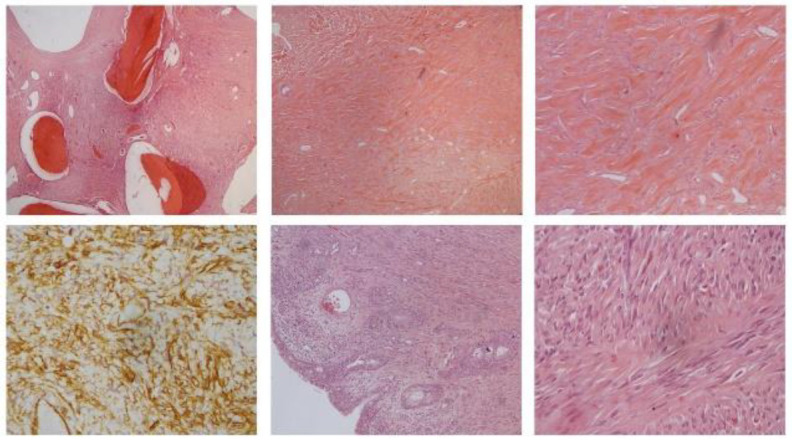
Histopathological pictures of the left sino-nasal solitary fibrous tumour showing the presence of bone entrapped by the tumour (top left). The presence of proliferation of spindle cells is seen under low power view (top middle) and thick collagen stroma (top right). Immunohistochemistry showing positive beta catenin seen in left sinonasal solitary fibrous tumour (bottom left). Left sino-nasal solitary fibrous tumour lined by respiratory type epithelium with the underlying proliferation of spindle cells (bottom middle). The presence of long fascicle cells is seen (bottom right)

Histopathological evaluation of the resected left sino-nasal mass (Figure 4), measuring 65mm in aggregate (with 25x15x3mm bony fragments) showed submucosal proliferation of spindle cells arranged in a vague sweeping fascicle embedded in a dense collagenous keloidal stroma. The spindle cells exhibited bland hyperchromatic nuclei with indistinct cytoplasm with interspersed staghorn-like and congested blood vessels. The tumour was seen entrapping and invading the bone fragments. Mitosis was scattered, less than 4 mitoses per 10 high-power fields were observed. No necrosis was seen. On immunohistochemistry, it showed positive CD34, bcl-2, vimentin and beta-catenin (patchy) and was negative for SMA, Desmin, Myogenin, S100, TLE-1, and CD56 with Ki67 positivity of about 2%. A final impression was in favour of a solitary fibrous tumour was given.

**Fig 5:**
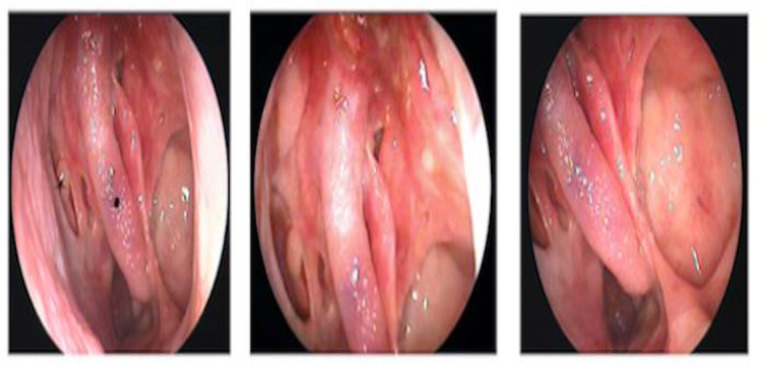
Clinical photograph of the endoscopic view of left nasal cavity one year postoperatively with a wide opening into paranasal sinuses; (1) star – posterior septal perforation, bullet – mucosal flap used to cover skull base defect, (2) well-epithelized skull base mucosae, (3) view of healthy maxillary sinus mucosa

Post operatively, she was reviewed on regular intervals with surveillance nasoendoscopies performed up to one year later. There was no clinical evidence of local recurrence, (refer to Figure 5) with MRI surveillance.

## Discussion

The solitary fibrous tumour (SFT) is a rare soft tissue neoplasm. It has been postulated that it is mesenchymal in origin rather than mesothelial due to its ubiquitous location (4). The reason behind the controversial histogenesis is because of most cases of solitary fibrous tumours are being reported in pleura (5). Therefore, it explained the theory of mesothelial origin. The same explanation goes for cases that occur in the mediastinum, pericardium and peritoneum due to the presence of mesothelium tissues or serosal surfaces. However, this theory does not explain the presence of this tumour in the central nervous system, head and neck region and extremities. 

 In a study done by Gold et al, solitary fibrous tumours of the head and neck region account for 6% with most cases reported in the oral cavity (2). Meanwhile, the nasal cavity and paranasal sinuses are extremely rarely affected. Generally, it can affect adults of a wide age range, ranging from 18 to 82 years old with a median age of between 49 to 58 years old reported in several studies. In addition, both males and females are equally affected by this disease. However, it is found that males are more affected by solitary fibrous tumours of paranasal sinuses and nasal cavities in some of the studies conducted (6).

 Most of the sino-nasal solitary fibrous tumours are generally described as slow-growing tumours, with a range of duration of symptoms from 0.25 to 24 months, with an average of 9.9 months (3). Duration of symptoms of 36 months has been reported as well. It mostly exhibits benign characteristics. Nevertheless, it is locally invasive, and its extension to the orbit and base of the skull has been reported (7). 

In addition, local recurrence with or without metastasis has been reported too. Thus, it is classified as a borderline or low-grade malignant soft tissue tumour in WHO Classification of Head and Neck Tumours 2017. To suggest it is an aggressive tumour, predictive factors of age more than 55 years old, tumour size more than 15cm, and more than 4 mitoses per 10 high-power fields are considered (8). In general, non-contrasted CT of paranasal sinus shows homogenous isoattenuating lesion (9) and showed marked homogeneous enhancement after administration of contrast (10). Bone remodelling, thinning, local absorption and reactive sclerosis can be seen, however, it is non-specific. MRI is another useful imaging in evaluating the tumour and its extension, especially intracranial extension. In T1 weighted images, solitary fibrous tumours are homogenously isoattenuation to grey matter, whereas they are heterogeneously isointense or hypointense in T2 weighted images (9). In addition, marked inhomogeneous enhancement can be observed in contrasted T1 weighted images (10), which is seen in this case as well. 

 Definitive management of this disease is complete surgical resection of the tumour. Several surgical intervention approaches had been reported, namely the endoscopic approach (10) and the open approach, for example, the lateral rhinotomy approach. Transcranial and transfacial approaches have been described in cases of solitary fibrous tumours with a base of the skull or intracranial extension (11). 

Certain considerations need to be taken into account in choosing the appropriate approach. In this patient, a combined endoscopic and open lateral rhinotomy approach is preferred due to the extensive extension of the tumour locally and intracranially. The main aim is to achieve a clear margin of the tumour and to prevent a recurrence. However, complete surgical excision might be difficult due to the close proximity of vital structures such as the orbit, dura and the vascularized nature of the tumour. Pre-operative embolization of the feeding vessel before the operation had been reported (11), to prevent excessive blood loss. An entirely endoscopic approach may be difficult to achieve complete tumour clearance, due to the piece-meal nature of resection. Overall, the outcome is good with complete excision with a low recurrence rate reported in several studies (3,6). In one of the 2 cases of sino-nasal solitary fibrous tumours reported by Dipak et. al, it was found that the patient had local recurrence twice and lateral rhinotomies were carried out twice to excise the tumours (7). 

Adjuvant therapies were also described with conventional chemotherapy and were reported to be effective in controlling or stabilizing locally advanced or unresectable and metastatic solitary fibrous of all anatomical sites (12). 80% of the patients achieve stable disease after receiving chemotherapy of all lines. Diagnosis of solitary fibrous tumours is mainly from histopathology and its immunohistochemistry. Typical microscopic findings of the tumour that is submucosal, pseudoencapsulated, and variably cellular, consisting of spindle-shaped cells arranged in a haphazard pattern. The vessels are stellate to staghorn-like in shape. There will be a variable collagenous background. Almost all cases of solitary fibrous tumours, regardless of anatomical location, will show diffuse positivity towards CD34 (13), a transmembrane glycoprotein found on the surface of hematopoietic progenitor cells. However, it is not a specific marker for solitary fibrous tumours as it can be found on variable types of mesenchymal cells (14). 

Therefore, this finding needs to be coupled with the findings of negative expression of other immunohistochemical markers, such as S-100 protein and desmin. Moreover, vimentin and bcl-2 positivity can be seen sometimes (6), but it is not specific too, as it is expressed by most mesothelial and many other epithelial tumours. STAT6, on the other hand, has gained popularity to become a more reliable marker in diagnosing solitary fibrous tumours nowadays (3). It is reported that it has a sensitivity of 95%. In a study done by Thompson et. al., it was found that all six patients showed strong and diffuse positivity towards STAT6.

## Conclusion

Solitary fibrous tumours (SFTs) are an uncommon type of sino-nasal neoplasms. The nature of the expansion of SFTs over time and the proximity of the region to the skull base and orbit, predilect an extension towards these structures. The diagnosis of SFTs may only be confirmed after a histopathological evaluation of the surgical specimen. 

Therefore, a comprehensive radiological assessment is necessary for determining the extent of the tumour for safe surgical management, with the subsequent need for close interval clinical surveillance to exclude recurrence.
